# “Acoltremon: a novel advancement in dry eye disease management”

**DOI:** 10.1097/MS9.0000000000004126

**Published:** 2025-10-17

**Authors:** Muddassir Khalid, Esha Zahid, Muhammad Shaheer

**Affiliations:** Department of Medicine, Nishtar Medical University, Multan, Pakistan

*Dear Editor,*

Dry eye disease (DED) is a complex and multifactorial disease of the ocular surface and is defined by tear film instability, ocular surface inflammation, and neurosensory abnormalities. It disrupts the general well-being and daily functioning of the patient as it manifests as chronic ocular discomfort and severe visual symptoms^[1,2]^. Even the well-established treatment options, such as artificial tear supplements, topical anti-inflammatory medications, and punctal occlusion, usually only provide partial or delayed relief from symptoms. The clinical limitation of treatments highlights that we need to focus on disease-specific therapies that target underlying mechanisms instead of focusing only on symptom management^[3,4]^. Acoltremon (AR-15512), also called Tryptyr, is the first topical TRPM8-mimetic agonist designed to activate corneal sensory nerves to boost the body’s natural tear production. This substance causes basal tear secretion in a natural way by activating TRPM8, a receptor for cold-sensitive ion channels. Findings from a phase 2b, randomized, vehicle-controlled clinical trial (COMET-1) showed that AR-15512 produced statistically significant improvements in the objective signs and patient-reported symptoms of DED. The participants of this clinical trial reported an improvement in the quality of life related to vision by this corticosteroid-sparing approach^[5]^. In contrast to currently available prescription anti-inflammatory therapies, such as topical cyclosporine and lifitegrast, which primarily deal with ocular surface inflammation and typically present with reduced patient satisfaction and a slow onset of effect and treatment duration, recent systematic reviews and meta-analyses articles on transient receptor potential (TRP) channel modulators (including TRPM8 agonists) validate the therapeutic value of this approach. A thorough evaluation shows that the regulation of the TRP channel is more secure and better tolerated. It promotes the health of the ocular surface and stimulates tear production at the same time^[6]^. This has raised the awareness of the potential benefits of neuromodulatory techniques that may help in quicker and more reliable recovery of neural-based tear production and ocular comfort arousal. When combined, the data from clinical trials and new translational research clearly point to Acoltremon as a potentially significant addition to the treatment arsenal for DED^[4]^. It is especially promising for patients with tear-deficient forms of the disease who need a quicker and more reliable restoration of ocular surface homeostasis because of its exceptional capacity to stimulate basal tear flow. Safety evaluations, clinical trials, and the use of Acoltremon in real clinical practice will further confirm these initial findings.

TITAN Guidelines: This manuscript complies with TITAN Guidelines, 2025, declaring no use of AI^[7]^.

## Data Availability

Not applicable.

## Peer and provenance statement

Not commissioned, externally peer-reviewed.
